# 0520. The role of mitochondrial dysfunction in the pathophysiology of icu-acquired weakness

**DOI:** 10.1186/2197-425X-2-S1-P29

**Published:** 2014-09-26

**Authors:** K Jiroutkova, J Ziak, A Krajcova, M Fric, V Dzupa, F Duska

**Affiliations:** Charles University, 3rd Faculty of Medicine, Laboratory of Bioenergetics, Prague, Czech Republic; FNKV University Hospital, Intensive Care Unit, Prague, Czech Republic; FNKV University Hospital, Dept. of Trauma and Orthopaedics, Prague, Czech Republic; Nottingham University Hospitals NHS Trust, Adult Intensive Care Unit, Nottingham, UK

## Introduction

Mitochondrial dysfunction (bioenergetic failure) is knownt to contribute to the development of multiorgan failure in acute sepsis. The defect is mainly at the level of respiratory complex I [1, 2].

## Objectives

To assess whether mitochondrial dysfunction in skeletal muscle perists until protracted critical illness and whether it contributes to the development of ICU-acquired weakness.

## Methods

Muscle biopsies were obtained from critically ill patients with severe ICUAW (defined as MRC score < 24 on two separate tests) and from otherwise healthy controls undergoing elective hip replacement. 50-100mg of the native sample was homogenized by a technique which perserves mitochondria in their cytosolic context [3]. Oxygen consumption was measured by high-resolution respirometry (Oxygraph-2Km Oroboros) under two occasions: 1. homogenate enriched with substrates was sequentially treated with an ATPase inhibitor (oligomycine), an uncoupler (FCCP) and KCN. This allowed us to measure oxidative phosphorylation, respiratory chain capacity, proton leak through inner mitochondrial membrane and non-mitochondrial O2 consumption. 2. skeletal muscle homogenate enriched with abundant ADP was treated with sequential addition of substrates and inhibitors of complexes I, II, III, and IV, respectively. Integrity of outer membrane was assessed by measuring the response to addition of cytochrome c. Oxygen consumption rate was normalized to citrate-synthase activity and total protein content.

## Results

In skeletal muscle of patients with protracted critical illness and severe ICUAW we have found the activity of complex II and III to be >200% of activity of healthy controls. Function of complexes I and IV was identical as were global mitochondrial function/integrity indices.

**Table 1:** Activities of respiratory complexes [pmol/nkat]

Data as median (IQR)	Complex I	Complex II	Complex III	Complex IV	Leak	Resp. chain spare capacity
ICUAW (n=6)	22 (16-23)	23 (20-28)	7 (4-8)	67 (62-93)	7 (5-11)	43 (40-46)
Controls (n=5)	21 (20-25)	9 (3-19)	3 (2-4)	59 (42-109)	8 (5-10)	45 (36-73)
p [Mann Whitney	0.860	0.045	0.028	0.715	0.806	0.724

Upregulation of complex II may represent an adaptive phenomenon as skeletal muscle during critical illness is more reliant on fatty oxidation (which feeds electrons to respiratory chain via complex II), because of insulin resistance and impaired oxidative glucose disposal.

## Conclusions

In patients with severe ICUAW we have demonstrated increased activity of respiratory chain complexes downstream to complex I and intact global mitochondrial function. This may represent an adaptation to insulin resistance.
